# Perioperative Management of Complex Hepatectomy for Colorectal Liver Metastases: The Alliance between the Surgeon and the Anesthetist

**DOI:** 10.3390/cancers13092203

**Published:** 2021-05-03

**Authors:** Enrico Giustiniano, Fulvio Nisi, Laura Rocchi, Paola C. Zito, Nadia Ruggieri, Matteo M. Cimino, Guido Torzilli, Maurizio Cecconi

**Affiliations:** 1 Department of Anesthesia and Intensive Care Units, IRCCS Humanitas Research Hospital, 20089 Milan, Italy; laura.rocchi@humanitas.it (L.R.); paola_cosma.zito@humanitas.it (P.C.Z.); nadia.ruggieri@humanitas.it (N.R.); maurizio.cecconi@hunimed.eu (M.C.); 2 Hepato-Biliary & Pancreatic Surgery Unit, IRCCS Humanitas Research Hospital, 20089 Milan, Italy; matteo_maria.cimino@cancercenter.humanitas.it (M.M.C.); guido.torzilli@hunimed.eu (G.T.); 3 Department of Biomedical Sciences, Humanitas University, 20090 Milan, Italy

**Keywords:** liver surgery, colorectal, hepatic resection, perioperative care

## Abstract

**Simple Summary:**

Major high-risk surgery (HRS) exposes patients to potential perioperative adverse events. Hepatic resection of colorectal metastases can surely be included into the HRS class of operations. Limiting such risks is the main target of the perioperative medicine. In this context the collaboration between the anesthetist and the surgeon and the sharing of management protocols is of utmost importance and represents the key issue for a successful outcome. In our institution, we have been adopting consolidated protocols for patients undergoing this type of surgery for decades; this made our mixed team (surgeons and anesthetists) capable of achieving a safe outcome for the majority of our surgical population. In this narrative review, we report the most recent state of the art of perioperative management of hepatic resection of colorectal metastases along with our experience in this field, trying to point out the main issues.

**Abstract:**

Hepatic resection has been widely accepted as the first choice for the treatment of colorectal metastases. Liver surgery has been recognized as a major abdominal procedure; it exposes patients to a high risk of perioperative adverse events. Decision sharing and the multimodal approach to the patients’ management are the two key items for a safe outcome, even in such a high-risk surgery. This review aims at addressing the main perioperative issues (preoperative evaluation; general anesthesia and intraoperative fluid management and hemodynamic monitoring; intraoperative metabolism; administration policy for blood-derivative products; postoperative pain control; postoperative complications), in particular, from the anesthetist’s point of view; however, only an alliance with the surgery team may be successful in case of adverse events to accomplish a good final outcome.

## 1. Introduction

Hepatic resection has been widely accepted as the first choice for the treatment of colorectal metastases [1]. Colorectal cancer often causes metastases to the liver (14–70% of patients), of which modern surgery combined with neo-adjuvant chemotherapy permits the resection both by open and laparoscopic surgery [2]. Hepatic surgery is considered a major surgery (MS); as such, it exposes patients to the risk of postoperative complications. Furthermore, given the increasing number of subjects who undergo high-risk surgery in old age along with age-related comorbidities, the risk of postoperative complications further increases.

Enhanced Recovery After Surgery (ERAS) programs ameliorated the perioperative course of major abdominal surgery. In particular, for digestive tract surgery, specific guidelines became available in the past decade (see www.erassociety.org, (Accessed on 2 February 2021) [3,4].

Modern surgery cannot ignore the need in a multimodal approach to prepare patients to high-risk surgery. The best way to ensure a safe outcome is to start making use of each specialist doctor who manages each aspect according to specific competences before the operation and continue this practice in the postoperative period.

Our review tried to take stock of the anesthetic aspects of hepatic resection without forgetting the potential surgical adverse events, focusing on the closest perioperative period as follows: (1) preoperative evaluation and examinations; (2) general anesthesia and intraoperative fluid management and hemodynamic monitoring; (3) intraoperative alterations of metabolism and their treatment; (4) administration of blood-derivative products; (5) postoperative pain control; (6) postoperative complications.

## 2. Preoperative Considerations

Preoperative assessment was adapted to individual patients and types of surgical resection. Young patients (<40 years) without underlying liver disease could undergo significant liver resection having the same preoperative work out of any major intra-abdominal operation. Conversely, patients with hepatic disease have an increased risk of intra- and postoperative complications and require an in-depth preoperative assessment.

During the past decades, the liver surgery-associated mortality has reduced to less than 2% in referral centers, but the rate of postoperative adverse events is still high (20–50%) [5,6,7]. To quantify the operation-related risk, many score systems have been used.

Despite the ASA (American Society of Anesthesiology physical status classification system) score being a simple tool adopted all over the world to evaluate patients preoperatively, it does not take into account the type of operation the patient undergoes. In 2014, the European Society of Cardiology/European Society of Anesthesiology (ESC/ESA) guidelines on non-cardiac surgery recommended a healthy lifestyle and the correction of unstable clinical conditions to make patients arrive to the surgical theater with a sufficient functional capacity [8,9].

The DASI (Duke Activity Status Index) should be a reliable system of evaluating and predicting the postoperative outcome, in particular, adverse cardiac events [10]. Finally, the surgical Apgar score (SAS), even though it is not so widely used, could be a complementary reliable and simple system for estimating the risk of a poor postoperative outcome [11].

At any rate, the preoperative period should ameliorate the starting clinical conditions of patients, aiming at better results of the perioperative period.

The ERAS Society recently released guidelines for fast-track management of patients undergoing liver surgery. The recommendations for the perioperative management of patients could be summarized as described below. (1) Recommended preoperative fasting of 6 h for solids and 2 h for liquids. Carbohydrate supplies may be used the evening before surgery and at least 2 h before anesthesia induction. (2) Short-acting anxiolytic drugs should be preferred over the long-acting ones to facilitate regional anesthesia prior to the surgery. (3) Administration of low-molecular-weight heparin (LMWH) or unfractionated heparin should start 2–12 h before the surgery aiming at reducing the risk of thromboembolism. (4) Minimally invasive procedures should be preferred where possible. (5) Maintenance of intraoperative normothermia. (6) Early oral intake at the first postoperative day and early mobilization (as soon as possible). (7) Routine epidural analgesia (EA) cannot be recommended for open liver surgery for ERAS patients. Wound infusion catheter or intrathecal opiates can be good alternatives combined with multimodal analgesia. (8) Prevention of postoperative nausea and vomiting (PONV). (9) Fluid management includes the central venous pressure (CVP) guide with a target < 5 cm H_2_O. (10) Steroids (methylprednisolone) may be used before hepatectomy in normal liver parenchyma since it decreases liver injury and intraoperative stress without increasing the risk of complications. (11) Glycaemia control [4].

Cardiac function evaluation should assess the ability of the system to cope with the hemodynamic challenge of vascular exclusion during liver resection. Exercise or stress echocardiography may be useful to assess the contractile reserve. Relevant anamnestic data are those regarding previous neo-adjuvant chemotherapy, which may reduce functional cardiac reserve, and/or conditions causing elevation of central venous pressure (CVP) that significantly increase the risk of intraoperative bleeding [1].

Pulmonary evaluation focuses on detecting impaired pulmonary gas exchange or inadequate ventilatory reserve. Pulmonary function testing and arterial blood gas analysis should be considered in case of abnormal values at pulse oximetry testing. A significant percentage of patients with cirrhosis or portal hypertension is affected by some degree of hepatopulmonary syndrome, producing varying degrees of hypoxemia depending on the degree of intrapulmonary shunting. Typical signs are platypnea (upright positional dyspnea), finger clubbing and spider nevi. In such cases, contrast-enhanced echocardiography is recommended for diagnosis, even if it cannot quantify the extent of shunting.

Hepatic reserve quantification is helpful in predicting the risk of postoperative liver failure. Currently, no single test reliably predicts postoperative liver failure, and the assessment is based on laboratory and radiological investigations, quantitative tests, and surgical judgment. Conventionally, in young patients (<40 yrs) with normal hepatic parenchyma, it is safe to remove up to 50–60% of the whole volume of the liver, whilst patients with chronic liver disease presenting for liver resection are at high risk of postoperative liver failure and require a more detailed assessment of the hepatic function. Indeed, cirrhosis limits the ability of the liver to regenerate. Finally, we should remember that it is contraindicated to operate patients presenting with obstructive jaundice or for emergency liver resection since these factors associated with the highest perioperative morbidity and mortality [12,13].

The Child–Pugh clinical scoring system (Table 1) has been used as a reliable validated prognostic tool for patients with chronic liver disease undergoing general or portocaval shunt surgery and has gained widespread use in hepatobiliary surgery, mostly in case of Hepatocellular carcinoma (HCC). It has recently been suggested that patients with scores of B or C should not receive liver resection surgery [14]. Further specialized testing of the hepatic function, such as assessment of indocyanine green (ICG) retention, is available, but its description goes beyond the scope of this article.

## 3. General Anesthesia Management and Intraoperative Hemodynamics

The anesthetic issues for this type of surgery are almost the same as for other major operations. General anesthesia is mandatory and short-acting drugs should be preferred. Needless to say, communication regarding surgical manipulations and management of hemodynamics between the anesthesiology and surgical staff is a critical component of achieving the optimal outcome.

Blood flows to the liver (around 1.5 L/min) through two vascular systems: 20% flows from the hepatic artery (HA), 80%—from the portal vein (PV). This amount of blood returns to the right side of the heart by suprahepatic veins that merge into the inferior vena cava just before it flows into the right atrium. Several blood loss-limiting surgical techniques could be adopted by the surgical team aiming at reducing blood loss, hemodynamic impairment, and transfusion-related complications. Hence, excluding hepatic circulation from systemic circulation during dissection and transection of parenchyma might be achieved by means of temporary hepatic inflow occlusion (Pringle maneuver) and total (inflow and outflow) vascular exclusion (TVE).

The Pringle maneuver (PM) consists of intermittent clamping of the hepatic hilum (HH): generally, 15–20 min of interrupted blood flow are followed by 5 min of reperfusion. The PM has been shown to provide a sort of protection of the liver tissue from hepatic injury due to the ischemia-reperfusion injury (I-RI) which should be inevitable for longer and non-intermittent occlusion of the blood flow, [1].

The most effective vascular control is obtained with total vascular exclusion (TVE). After performing the Pringle maneuver, clamps are applied sequentially across the infra-hepatic IVC above the renal veins and across the suprahepatic IVC.

Intuitively, with this technique, hemodynamic instability is likely and potentially profound, with venous return decreasing and systemic vascular resistance increasing, and requires aggressive hemodynamic management [13].

At our hospital, the PM is almost always chosen as the main option. The intermittent interruption of the blood flow makes the cardiac afterload and preload vary coherently. After the HH clamping, the afterload can increase by 20–30% and cardiac output (CO) could fall up to 10%. The PM also aims at limiting the blood loss. When necessary, the ultrasound-guided finger compression of the right hepatic vein by the surgeon increases the effectiveness of the PM to control backflow bleeding [15].

Although evidence of the clinical benefit of administration of N-acetylcysteine (NAC) to limit the I-RI remains contradictory, several studies have reported the effectiveness of NAC (15 mg/kg i.v. bolus) to help cells to metabolize free radicals that are released during the ischemic phase [16,17,18,19,20].

**Figure 1:**
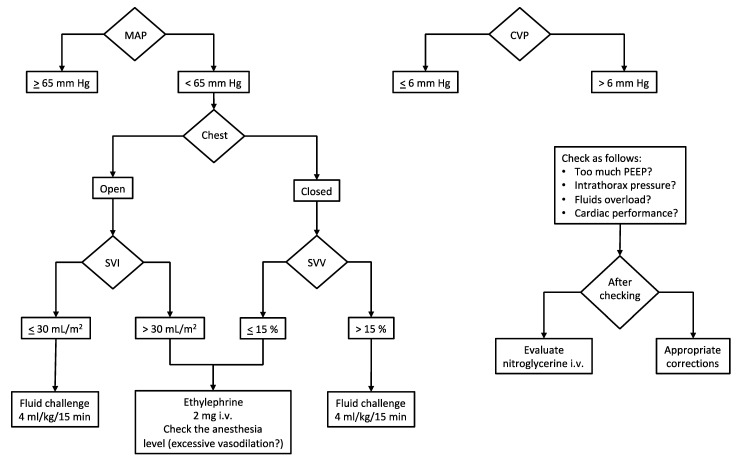
Intraoperative hemodynamic management. Our institutional algorithm for intraoperative hemodynamic management based on target MAP and CVP. Preload dynamic indices (or stroke volume indices) are used to assess fluid responsiveness and guide vasopressor administration. Abbreviations and units. CVP, central venous pressure (mm Hg); MAP, mean arterial pressure (mm Hg); PEEP, positive end-expiratory pressure (cm H_2_O); SVI, stroke volume index (mL/m^2^); SVV, stroke volume variation (%).

Given the above, it is of utmost importance to adopt hemodynamic monitoring during the surgery (Figure 1).

Beyond the standard monitoring (invasive blood pressure, IPB; electrocardiogram with heart rate, HR; end-tidal CO_2_, EtCO_2_; peripheral oxygen saturation, SpO_2_), the most important parameters which deserve to be measured are cardiac output (CO), stroke volume (SV) and stroke volume variation (SVV) when open-chest surgery is not needed. Since the SVV depends on the interaction between the heart and the lungs (i.e., intrathoracic pressures), this parameter is not reliable in case of the open chest. Conversely, when the chest is closed, the intermittent rising of the intrathoracic pressure (due to positive pressure ventilation) limits the venous return and, consequently, the cardiac stroke volume cyclically. Such continuous variation of the SV is called stroke volume variation and is expressed as a percentage. When SVV is higher than 13–15%, it means that the SV and the CO can be increased by the fluid load (i.e., fluid responsiveness) [21].

Safe cardiocirculatory assessment considers target hemodynamic values as follows: CVP, 0–6 mm Hg; CI, 2–3 L/min/m^2^ of the body surface area (provided the stroke volume index > 30 mL/m^2^ of the body surface area in subjects with normal cardiac ejection fraction, >55%); SVV, 10–15% during the resection phase (a value of SVV ≤ 10% could be accepted once liver dissection has concluded).

A peculiar issue regards the monitoring of central venous pressure (CVP). There is a general agreement on its maintenance below 6 mm Hg to limit the backflow bleeding due to the suprahepatic veins’ increased resistance in case of high pressure into the right atrium during the resection phase. Hence, a reduced venous distension ensures reduced hepatic bleeding. In addition to this, facilitated mobilization of liver and dissection of hepatic veins are observed. In consideration of findings of improved outcomes, several authors have advocated widespread adoption of the low CVP approach in hepatic resection [15]. Our protocol, aiming at reducing the CVP, includes a limitation of fluid input (4–6 mL/kg/h of balanced crystalloids) along with nitrate infusion (if necessary) to reach the target right atrial pressure. Furthermore, a protective mechanical ventilation strategy (tidal volume of 4–6 mL/kg and positive end-expiratory pressure < 5 cm H_2_O) contributes to maintaining the CVP in the low range (see Section 3.2).

Although ERAS protocols recommend CVP monitoring for fluid therapy management, we should point out that right atrial pressure (i.e., CVP) represents a marker of how the heart manages the venous return. Therefore, CVP should be taken into account more as a marker of cardiac ability to receive and eject blood forward than as a measurement of volume assessment [22].

Serum lactate (sLac) plays an important role not only because its clearance depends on the liver function, but also because lactatemia is a marker of tissue perfusion and, consequently, an indirect measurement of impaired cellular metabolism [23,24]. During the PM, sLac is expected to rise in a way directly proportional to the single and complete duration of the HH clamping. Hence, it is also intuitive that in case of a low-flow state, a further amount of lactic acid is released into the blood stream due to the cellular metabolism switching into the anaerobic way because of lower oxygen delivery (DO_2_). Serum lactate (sLac) concentration depends on the balance between production and clearance from the blood stream, and it has been reported to be a predictor of outcome in critically ill patients, including those with liver failure, sepsis and trauma [23,24,25,26,27,28,29,30,31]. Although the peak serum concentration of lactate may correlate with the outcome, its clearance (cLac) seems to be a better predictor [32,33,34].

Hence, we can see how important the continuous hemodynamic assessment is as it aims at avoiding cardiocirculatory imbalance which could affect the postoperative outcome as we found in a retrospective analysis of our database including more than 300 patients forwarded to hepatic resection surgery. In a previous retrospective study, we observed how the intraoperative fluid regimen and the outcome ameliorated after we started monitoring the CO, even when using a semi-invasive method, in the patients forwarded to liver resection [35,36].

### 3.1. How Much Fluid Does a Patient Need?

The ERAS (Enhanced Recovery After Surgery) protocols recommend that intraoperative fluid load should be limited. At the same time, an excessively restrictive fluid regimen (also in order to limit the intraoperative backflow bleeding) may cause a low-flow state. Modern hemodynamic monitoring based on the heart–lung interaction can predict the response of the patient to fluid administration. It depends on the point of the Frank–Starling curve where the heart of the patient is: at the steep portion of the curve, it will respond positively (i.e., SV increases by 10%); conversely, at the flat portion of the curve, it will not respond. According to this new concept of “functional hemodynamics”, we can predict such a response observing the so called dynamic indices, the most used of which are SVV and PPV (pulse pressure variation, i.e., the percentage of variation of the pulse pressure, that is the difference between systolic and diastolic blood pressure). Since the prediction of fluid responsiveness (FR) obeys the rules of heart–lung interaction caused by the inverted intrathoracic pressure during mechanical ventilation when the surgeon has to open the chest in order to better isolate the liver, the dynamic indices are not reliable. In that case we can observe the stroke volume and the cardiac output to assess hemodynamics [36,37,38,39,40,41]. Last but not least, particularly both during the isolation of the liver and the resection phase, surgical manipulations often cause a stretching of the IVC which reduces the venous blood return and consequently the reduction of the SV and CO. The measurement of the diameter of the IVC by ultrasound performed by the surgeon in the operative field does not help to solve the question of the IVC diameter reliability and helpfulness in fluid management [42].

### 3.2. How to Manage CVP?

In our opinion, the more the inferior vena cava is “filled”, the less its displacement will cause hemodynamic impairment. The challenge is to find the optimal compromise between fluid overload and an “empty” IVC. The whole integration of the parameters we measure and monitor could address this issue. Our fluid management protocol includes a limitation of fluid input by no more than 4–6 mL/kg/h of balanced crystalloids during the pre-transection and resection phases of surgery. After the resection of the hepatic parenchyma, fluid input is permitted to a limit of 7 mL/kg/h until the patient awakening in the recovery room. Then, the postoperative fluid input consists of 60–80 mL/h as necessary.

Moreover, aiming at giving the appropriate amount of fluids without making the CVP rising (target < 6 mm Hg), nitroglycerine i.v. infusion may be an option. At the same time, since it may provoke hypotension, norepinephrine can guarantee a mean arterial pressure (MAP) > 65 mm Hg and changing the dose rate according to the clamping and unclamping of the hepatic hilum.

Finally, a protective mechanical ventilation strategy (tidal volume of 4–6 mL/kg and positive end-expiratory pressure ≤ 5 cm H_2_O) avoids excessive rising in intrathoracic pressure and consequently minimizes impact on CVP increase.

## 4. Intraoperative Acid–Base Balance and Metabolic Issues

The anesthetic management during partial hepatic resection represents a challenge from a metabolic point of view as well. Indeed, the liver is one of the most important organs responsible for detoxifying metabolites, synthesizing proteins and producing biochemical substances involved in homeostasis.

Our goals for liver resection are to avoid the increase of preexisting hepatic disorders and preserve function of the residual liver parenchyma as it may have important effects on the postoperative course in terms of mortality and morbidity [43].

Three aspects need to be considered: (1) patients undergoing liver resections may have different levels of hepatic dysfunction; (2) during parenchymal transection, the surgeon temporarily occludes the inflow to the liver to reduce blood loss (Pringle maneuver) causing hepatic ischemia; (3) liver function changes after resection because of decreased parenchymal volume.

These factors contribute to a variety of metabolic derangements, the most significant of which is lactic acidosis responsible for an increased anion gap and therefore for a progressive worsening of the acid–base balance.

Lactic acid (Lac) is a byproduct of anaerobic metabolism during tissue hypoxia. Normally, Lac is metabolized by the liver (that accounts for up to 50–70% of the whole lactate clearance (cLac)), while the kidneys clear approximately 30–33% and skeletal muscles do the rest. Moreover, serum lactate is an early indicator of impaired tissue microcirculation and it is associated with the outcome as serum Lac > 4 mmol/L persistent after 24 h is associated with a reduced survival in critically ill patients [44]. May a “hepatic-surgery patient” be similar to a critical care patient? We think so.

Chronic liver disease exacerbates hyperlactatemia in general, and this condition could already exist in patients affected by hepatocarcinoma or treated with chemotherapy that induces steatosis. Moreover, during partial hepatectomy with the Pringle maneuver, the liver reduces cLac and becomes itself a lactate producer. The resulting hyperlactatemia determines the occurrence of acidemia that, with concomitant hypoxia, definitely impairs lactate clearance, closing a vicious cycle. Furthermore, hyperlactatemia may be responsible for the reduced cardiac contractility and cardiac output and increased risk of arrhythmias. Intraoperative stresses, blood loss, endogenous release of stress hormones and vasoactive drugs administration may worsen the acid–base balance because these factors increase the amount of pyruvate which is converted into lactate. Serum lactate can also be increased by transfusion of stored blood, which contains some part of lactate, depending on the duration of storage [44].

Assuming appropriate ventilation in intubated patients during hepatic resection, metabolic acidosis can be mild (pH 7.30–7.36), moderate (pH 7.20–7.29) or severe (pH < 7.20). A reduction in extracellular pH alters the opening of proton-gated K^+^-channels in the myocardium and in blood vessels (along with nitric oxide release), enhancing arrhythmogenicity and contributing to their vasodilatation. Moreover, the opening of pH-gated potassium channels promotes apoptosis directly or acting in combination with hypoxia [43,44].

Normally, the rise in Ca^2+^ during acidemia contrasts the depressive effects of acidosis on the cardiac function. When we administer bases to counteract acidosis, we reduce ionized calcium levels and it may limit the improvement of the cardiac function despite the amelioration of acidosis. For this reason, it can be worthwhile infusing calcium during the operation [45].

**Figure 2:**
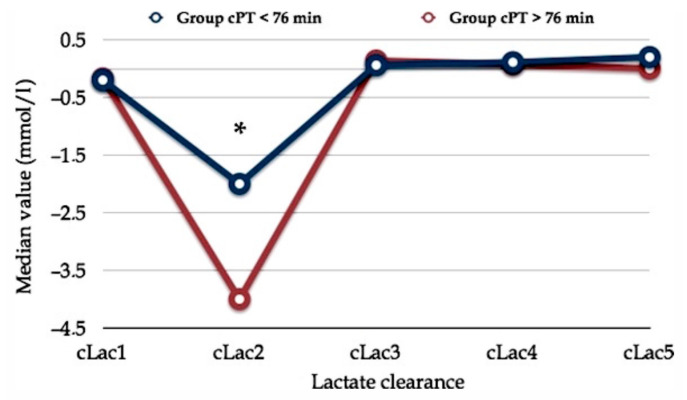
Early postoperative serum lactate clearance. The horizontal axis shows serum lactate clearance (cLac) at different postoperative hours (e.g., cLac1, cLac at the 1st postoperative hour; cLac2, cLac at the 2nd postoperative hour; etc.). The vertical axis shows the relative value of lactate clearance (cLac). Lac clearance was computed using the following formula: (sLac1 – sLac2)/sLac1. Two populations with different Pringle maneuver durations (cumulative Pringle maneuver time, cPT) are plotted. Asterisk (*) means statistical significance, *p* < 0.05. Comment. Reduced cLac and serum lactate accumulation produce the initial drop into the cLac curve. Serum lactate levels normalize following an increase in effective lactate clearance after the third postoperative hour. These finding persuades us to routinely wait three hours after the awakening of the patient before deciding whether the patient requires postoperative surveillance in the ICU. **Abbreviations. cLac**, serum lactate clearance; cPT, cumulative Pringle maneuver time. Reprinted with permission from reference [46].

In a previous report, we found a peculiar trend of early postoperative serum lactate concentration. Whatever the PM duration (more or less than 76 min), the postoperative clearance of serum lactate exhibited a particular curve shape that we named the “square root” shape as it draws a line which reminds us of the mathematical sign of the square root (Figure 2) [46].

In any case, perioperative management of lactic acidosis, especially when associated with severe acidemia, is an inseparable part of anesthesia, postoperative and critical care management since it presents a variety of challenges. It is of utmost important to maintain adequate organ perfusion, considering that liver capacity for lactate clearance is surely compromised by the ischemic phase due to the Pringle maneuver. This is even more essential if we consider that all general anesthesia techniques in the absence of surgical stimulation reduce the hepatic blood flow by about 30% [43].

In a large multicentric prospective study, Vibert et al. showed the prognostic value of end-surgery serum lactate concentration > 3 mmol/L as an independent predictive risk factor of postoperative complications. Hence, it can help clinicians regarding the need for intensive care unit admission after surgery. In addition, diabetes, repeated hepatectomy, major hepatectomy, major associated procedures besides hepatectomy, inflow occlusion and blood transfusion were the perioperative factors predicting increased lactate concentration [47]. Accordingly, Wiggans et al. concluded that the initial postoperative lactate concentration is a useful predictor of the outcome. In particular, patients with early postoperative serum Lac > 6 mmol/L exhibited a higher 90-day mortality than patients with serum Lac < 2 mmol/L (28% vs. 0.7%, respectively) [48].

To the best of our knowledge, there is no agreement about when, how and how fast lactic acidosis correction should be reached. Given the severity of prognosis linked to a lasting metabolic acidosis, an early cause-oriented treatment is crucial. The goal must be the normalization of blood lactate levels, pursuing the hemodynamic optimization, then tissue perfusion improvement and finally the restoration of proper lactate removal [47].

The use of sodium bicarbonate is simple and inexpensive. It was a cornerstone for acidosis treatment, but its administration in lactic acidosis is not effective and no benefit has been found in terms of clinical outcomes or mortality [49]. The use of sodium bicarbonate in this context is not recommended, but it may be beneficial in cases of severe acidemia in patients with renal failure or complications of advanced liver disease [43,44]. An alternative may be the administration of dichloroacetate. Unfortunately, dichloroacetate is metabolized almost exclusively by the liver in a way involving pyruvate dehydrogenase (PDH). Since in patients with cirrhosis the activity of PDH is reduced, it may be ineffective. Another option may be tromethamine (THAM) as it buffers protons thanks to the ammonia moiety improving the buffering capacity of the blood bicarbonate system. Its dosage is computed using the following Equation (1):

THAM (0.3 M) requirement (mL) = body weight (kg) × base deficit (mEq/L) × 1.1
(1)

It should be used with caution in patients with impaired renal function (GFR < 30 mL/min) because it would have limited efficacy in proton removal and retention of tromethamine in extracellular fluid may lead to hyperosmolality [43,44].

Finally, a helpful strategy to manage metabolic acidosis during partial hepatic resection consists of increasing the respiratory excretion of CO_2_. In intubated patients, we can modulate the ventilatory support increasing the respiratory rate or modifying the tidal volume aiming at reducing pCO_2_.

## 5. Blood-Derivative Products

During the past decade, the outcomes after liver surgery have improved progressively due to the surgical technique along with better knowledge of the hepatic anatomy, a more controlled intraoperative bleeding and a better perioperative management of patients.

During the 2018 consensus conference in Frankfurt (in line with the World Health Organization indications), the patient blood management (PBM) protocol focusing on evidence-based medicine (EBM) and patient safety in a systematic way was defined in order to ensure a good outcome. The protocol optimizes blood transfusion trying to minimize the excessive exposure to the risk of post-transfusion adverse events. However, there is no strong evidence about the threshold of serum hemoglobin (Hb) at which transfusion should be recommended, even in major hepatobiliary surgery [50].

In 2019, the Canadian Hepato-Pancreatic-Biliary Association group released a declaration that obtained 70% of consensus about the following recommendations: (1) preoperative evaluation of the risk of transfusion; (2) preoperative administration of iron in case of anemia; (3) restrictive fluid protocol during the clamping of the HH in order to limit the blood loss; (4) general perioperative restrictive transfusion strategies [51]. In the same year, the Liver Intensive Care Group of Europe (LICAGE) addressed the issue of the PBM in chronic liver disease as follows: (1) correction of preoperative anemia; (2) FFP administration only in case of bleeding; (3) in case of bleeding, platelet (PLT) administration if platelet count <50,000/mm^3^; (4) prothrombin time (PT) and time of partial thromboplastin activation (aPTT) should not be used to guide blood transfusions; conversely, viscoelastic tests are recommended; (5) in the course of bleeding, if fibrinolysis is ruled out and PLTs and fibrinogen are restored, prothrombin complex concentrates (PCCs) could be considered [52].

Restrictive strategy about the transfusion of red blood cells (RBC) has been demonstrated to be associated with a better postoperative outcome. Blood transfusion should replace only 60–70% of the blood loss. In particular, Makuuchi et al. adopted the cut-off value of hematocrit: 30% intraoperatively and 20% postoperatively as a threshold to start blood transfusion [52,53,54,55,56,57].

To the best of our knowledge, there is no general agreement about FFP transfusion, in particular, regarding its prophylactic use. In line with previous reports, we usually adopt the aPTT > 16–18 s as a cut-off marker to start FFP administration [58,59].

FFP administration is more frequent in Japan than in Europe and the United Sates. The cause of this routine use is not recommended by the Japanese Ministry of Health, but de facto 75% of patients with hepatocellular carcinoma (HCC) are affected by cirrhosis, too, and consequently receive FFP intraoperatively to sustain the coagulation system, to maintain the plasmatic osmolarity and reinforce the immune system to counteract the infective risk. Conversely, some trials found opposite results about the prophylactic use of FFP. Anyway, plasma is recommended if the international normalized ratio (INR) is >1.85. Several reports pointed out that another criterion for FFP administration may be the serum concentration of albumin (sAlb) 48 h after surgery. In particular, some authors recommend FFP if sAlb is lower than 2.4 g/dL [60,61,62,63]. Our institutional protocol requires restoring serum albumin also by the administration of human albumin 20%.

Finally, a red cells saver has been employed during surgery aiming at reducing heterologous blood consumption. In this case, leukoreduction is obtained by a specific filter and it is recommended over irradiation before reinfusion because of safety concerns with hypothetic tumoral spread [64].

**Figure 3:**
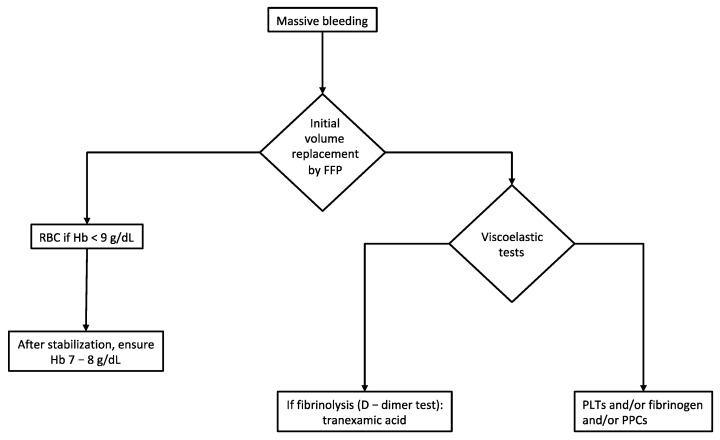
Decision algorithm for blood products administration. In the event of massive intraoperative bleeding (>1000 mL in less than 30 min or ongoing blood loss >150 mL/min), we agree with the surgical team to administer fresh frozen plasma (FFP) to sustain the volume. Serum hemoglobin of 7–9 g/dL is chosen as a cut-off value before starting red blood cells administration depending of patient comorbidities (preoperative anemia, cardiac status, etc.). Regarding pharmacological bleeding control, we adopt viscoelastic tests (e.g., ROTEM) as a point of care along with standard laboratory tests. Tranexamic acid, pooled platelets, fibrinogen and prothrombin complex concentrates (PCCs) are administered if coagulation tests show fibrinolysis, low platelet count (<100,000/mm^3^), hypofibrinogenemia and/or deficit of factors II, VII, IX, X, respectively. Temperature control and acidosis are an additional essential issue to be addressed in the event of hemorrhagic shock. Abbreviations. FFP, fresh frozen plasma; Hb, Hemoglobin; PLTs, platelet count; PPCs, prothrombin complex concentrates; RBC, red blood cells.

Figure 3 summarizes a proposal for the decision algorithm for blood products administration used at our institution. Pharmacological bleeding control is guided based on viscoelastic tests (e.g., ROTEM) as a point of care along with standard laboratory tests [65].

## 6. Postoperative Analgesia

Given the technical complexity and the potential occurrence of adverse events in hepatic surgery, the optimal postoperative pain control must be ensured in order to avoid further issues to an already high-risk operation. Hence, effective postoperative pain management is imperative.

The most common method for postoperative pain control is epidural analgesia (EA) [66]. When compared with systemic opioid analgesia, EA has consistently demonstrated superior pain control effectiveness, fewer respiratory complications, faster time to mobility and increased patient satisfaction [66,67]. In this chapter, we briefly explored advantages and disadvantages of EA utilization.

The benefits of epidural analgesia need to be weighed against its potential risks (i.e., epidural hematoma) [68]. Successful placement of an epidural catheter depends upon the experience of the operator, the anatomical characteristics of the patient and may be time-consuming.

The utilization of epidural catheters in patients undergoing open hepatic surgery is controversial. The main concerns are the following: (1) postoperative coagulopathy; (2) analgesic efficacy in comparison to other modalities of pain control; (3) hypotension that may require increased fluid administration with the risk of organ dysfunction [69]. Generally, in this type of surgery, the catheter should be inserted at a lower thoracic level.

Patients undergoing liver resection may have a deranged coagulation cascade, including thrombocytopenia, prothrombin time (PT) prolongation and increased international normalized ratio (INR), particularly between the first and fourth postoperative day, and in some cases, this can extend up to seven days. Postoperative coagulation derangement depends on several factors: the preexisting liver function, cirrhosis, preoperative platelet count and INR, volume of liver resection and the post-surgery remnant hepatic volume and function, intraoperative blood loss > 1 L, prolonged and complex surgery and ischemia-reperfusion injury [70].

Postoperative coagulation impairment and coagulopathy may occur. Following the ERAS Society’s recommendations, we do not routinely adopt epidural analgesia in liver resection due to concerns regarding safe removal of the catheter. In case of coagulopathy, delaying the removal of the catheter and/or the administration of fresh frozen plasma and/or platelet transfusion should be considered. In a retrospective study of 141 patients, 32% of patients received vitamin K or fresh frozen plasma to correct the INR to <1.3 before the removal of the epidural catheter. Moreover, it is worth remembering that accidental removal can occur in about 7% of cases and may expose the patient to the risk of undesired epidural hematoma [3,71,72].

In chronic liver disease, including cirrhotic patients, there is a complex interplay of procoagulant and anticoagulant factors rather than a derangement in INR and low platelets. We may observe an increase in factor VIII and the von Willebrand factor (vWF) which increase the adhesiveness of platelets to the endothelium [73]. Simultaneously, there is a decrease in ADAMTS-13, a liver-derived protease that cleaves the vWF into smaller and less sticky multimers that further increase platelet adhesiveness. Furthermore, the serum thrombin level is higher than normal; that, along with the deficiency of protein C, determines hypercoagulability and the prothrombotic setting [74,75,76]. All these coagulation impairments are against the utilization of EA, despite the fact that this technique attenuates the stress response to surgery and reduces the length of mechanical ventilation and postoperative ICU stay. Compared to general anesthesia alone or postoperative intravenous analgesia, EA has been found to attenuate postoperative immunosuppression [77,78]. In addition, it has been shown to be associated with reduced postoperative pulmonary complications and a lower rate of cardiac complications and it favors the recovery of gastrointestinal functions as it permits early mobilization of patients [77,79,80].

The sympathetic blockade and vasodilation due to thoracic epidural anesthesia may aid to maintain low central venous pressure (CVP) contributing to reducing backward blood loss. On the other hand, hypotension could occur and lead to an increased need in fluids both intraoperatively and during the postoperative period along with the risk of acute kidney injury (AKI) and other organ disfunction [79,81].

Alternatively, postoperative pain control may be assured by intravenous patient-controlled analgesia (PCA). Several randomized controlled trials have been carried out to compare the effectiveness and safety of PCA to epidural analgesia in patients undergoing open hepatic resection and have reported conflicting results [82,83]. In a systematic review and meta-analysis, Li et al. reported that EA was more effective than PCA in controlling postoperative pain in patients undergoing open hepatic resection, with no significant differences in length of hospital stay, complications or transfusion requirements [84].

More recently, alternative techniques have become more popular, for example, despite conflicting opinions on it, continuous wound infiltration (CWI) is spreading widely [83,85]. The comparison of CWI versus EA in liver surgery (LIVER trial) showed a faster recovery time in the CWI group, but EA provided a better early postoperative pain control [86]. Given the above, CWI should be considered as the first-line analgesic modality after liver resection when EA is contraindicated.

Finally, ancillary techniques deserve mentioning. Transversus abdominis plane (TAP) block and erector spinae plane (ESP) block, especially for laparoscopic surgery, should be considered complementary with a multimodal approach. Zhang et al. showed that ultrasound-guided TAP block with ropivacaine provides postoperative analgesia in laparoscopic hepatectomy. The concentration of ropivacaine associated with neurotoxicity was almost never reached [87].

Yassen et al. compared fentanyl PCA and TAP block with bupivacaine in cirrhotic patients undergoing liver surgery. Both groups exhibited effective pain control at rest (visual analog scale, VAS < 3), but on movement, bupivacaine provided a better effect [88].

In conclusion, a multimodal approach improves pain control and reduces opioid demand. At our department, in consideration of the potential post-surgery coagulation derangements, we routinely avoid the insertion of an epidural catheter.

Before the incision, we performed bilateral TAP block under ultrasound guidance or bilateral ESP block with a mixture of ropivacaine 0.375% + lidocaine 1%. As ancillary methods, in our experience, both are effective for postoperative pain control, also in case of a thoraco-phreno-laparotomic approach, along with paracetamol (40 min before the end of surgery) and morphine 0.1 mg/kg few minutes before the awakening from general anesthesia. Then, PCA (morphine 0.6 mg/mL at a rate of 2.1 mL/h) starts permitting self-managed rescue administration as follows: bolus 1.2 mg; lockout, 30 min. In addition, paracetamol 3 g/day i.v. (if not contraindicated) and NSAIDs (non-steroidal anti-inflammatory drugs) as rescue therapy are indicated no more than three times a day. The acute pain service allows us to regularly follow the patient postoperatively, detect and record the intensity of pain and consequently modify the therapy as appropriate.

## 7. Postoperative Complications

Hepatic surgery represents the gold standard treatment for colorectal liver metastases (CLM) [89]. Evolution of surgical techniques together with the use of new chemotherapies has permitted extending indications and increasing the complexity of surgical procedures, with a concomitant high risk of postoperative complications, [90]. The Clavien–Dindo classification (CDC) is a standardized system for the registration of surgical complications and its use has been validated and accepted worldwide in many fields of surgery [91]. In hepatic surgery, the incidence of complications varies from 20–50%, [92].

From a surgical point of view, the most common complications in order of frequency are bile leaks (post-hepatectomy biliary fistula, PHBF), post-hepatectomy liver failure (PHLF), post-hepatectomy hemorrhage (PHH), incisional infections, intraabdominal infection and major bleeding.

Other significant non-surgical complications impacting mortality are liver or renal dysfunction, respiratory failure and systemic sepsis. Main predictors for the development of postoperative complications include the ASA classification, age, extent of resection and simultaneous extrahepatic resection, perioperative blood transfusion and preoperative cirrhosis [1,12].

PHBF represents the major cause of postoperative morbidity [93]. Bile leaks increase the length of hospital stay, require prolonged abdominal drains, additional diagnostic imaging and/or therapeutic interventional procedures. In more severe cases, bile leaks could cause patient’s death due to the development of peritonitis, sepsis or liver failure [94]. Using the definition of the International Study Group of Liver Surgery (ISGLS), the incidence of PHBF ranges from 4.4% to 27.2% [95,96]. Spetzler et al. reported that in patients who required neoadjuvant chemotherapy, liver fragility and major hepatectomy are predicted risk factors for PHBF [97]. Remarkably, these findings are consistent with previous studies [98,99,100]. At our center, we developed a valuable tool for a new classification which allows detecting clinically relevant PHBF [101].

PHLF is defined as acquired deterioration of the ability of the liver to maintain its synthetic, excretory and detoxifying functions. It is characterized by an increased INR and hyperbilirubinemia on or after postoperative day 5 [102]. Nowadays, more patients can be at risk of PHLF due to the increasing prevalence of parenchymal liver diseases such as cirrhosis, non-alcoholic fatty liver disease and chemotherapy-associated liver injury (CALI), along with complex liver resection [103]. Even if there is no consensus on the association between CALI and PHLF, in our clinical practice, we use a cut-off of 40% of future remnant liver in patients treated with chemotherapy to predict PHLF [104]. For this reason, all the patients undergo a virtual cast 3D reconstruction of the planned surgery to predict the future remnant liver (FRL) [105].

Regarding post-hepatectomy hemorrhage (PHH), a standardized definition has not yet been established. The International Study Group of Liver Surgery (ISGLS) proposed a new definition and grading [106]. The definition of PHH depends on the value of postoperative serum hemoglobin in any postoperative transfusion of packed red blood cells (PRBC) or on the evidence of intraabdominal bleeding obtained by imaging and/or blood loss via abdominal drains. According to the literature, PHH is a quite rare event and in dedicated centers, its standard rate is below 3% [107].

The incidence of incisional infection has been reported to be up to 30.9% in hepatobiliary surgery [108]. It may appear as swelling and exudation at the incision site or, in the case of severe infection, dehiscence of the wound may be seen [109].

Finally, postoperative pulmonary complications are associated with significant morbidity and are mainly represented by pleural effusion, pneumonia and pulmonary embolism [110]. Despite the extensive use of the thoracoabdominal approach, in our clinical practice, these two latter events are very infrequent (depending on adequate antibiotic therapy, respiratory physiotherapy and thromboembolism prophylaxis with LMWH without forgetting the intraoperative protective ventilation strategy) [111,112,113]. Accordingly, most of the patients can be extubated at the end of surgery and be managed in a ward or the ICU depending on the risk of postoperative hepatic disfunction and respiratory impairment.

General postoperative principles of patient management include prevention of hypovolemia, early postoperative oral or enteral nutrition, glycemic control and hepatic function test monitoring, correction of electrolytes and coagulation abnormalities.

Fluid status should be monitored. Patients are at risk of both hypovolemia (patients may develop self-limiting ascites which can cause hypovolemia) and extravascular lung water accumulation due to reduced intravascular oncotic pressure and secondary hyperaldosteronism which promotes sodium and water retention. Restriction of sodium intake and diuretics to treat edema and ascites may worsen hypovolemia. Albumin 20% should be considered as a volume expander solution if needed [20].

The role of chemotherapy-associated liver injury (CALI) in the development of postoperative complications is still controversial. Some authors have reported no effect on postoperative outcome, some others observed an increase of morbidity and mortality associated with CALI [114,115].

Most patients can eat normal food on day one after surgery. Enteral or parenteral nutrition should be reserved to selected patients with ileus, delayed gastric emptying or malnourishment. Moreover, impaired hepatic mobilization of glucose could be an issue due to insulin resistance and hypo- or hyperglycemia. Glycemic control is mandatory, and it should be targeted through insulin or glucose infusion as needed [4]. Increase in hepatic transaminase and alkaline phosphatase levels is common and transient; nevertheless, a persistent elevation may lead to hepatic ischemia [1].

Major complex hepatic resection is an operation which highly exposes the patient to the risk of postoperative organ disfunctions. It has been established that postoperative hepatic failure prevention and diagnosis remain a significant challenge.

The regenerative ability of the liver is significant, and in a few days there is an increase in blood supply that ensures rapid tissue growth within the remnant liver. A hyperdynamic state with increased cardiac index and augmented splanchnic blood flow develops and persists for at least three days postoperatively, [116]. Monitoring of clinical and laboratory parameters gives a hint on true restoration of the functional ability of the liver parenchyma following increased blood supply. Urea levels may also be useful in diagnosing early incoming liver dysfunction.

Deteriorating liver function and unexplained neurological symptoms should prompt early diagnosis of encephalopathy, which may be supported by measurement of blood ammonia concentration. Hypoglycemia and postoperative drowsiness or confusion must be ruled out. Treatment is supportive and lactulose via nasogastric tube is useful to prevent gut stasis that contributes to encephalopathy.

Transient renal dysfunction is common. Reported incidence of postoperative AKI after liver resection ranges from 8 to 12% [78,80]. Major liver resection, blood transfusion, preexisting renal dysfunction and diabetes are some of the known risk factors for postoperative AKI. In addition, epidural analgesia and associated hypotension in the face of low CVP can increase the risk of AKI. To date, the most effective preventive measures are maintenance of normovolemia and adequate renal perfusion pressure [117].

Among electrolyte disturbances, hypophosphatemia is encountered in nearly all patients after major hepatic resection. It is hypothesized to be secondary to excessive urinary loss stimulated by phosphaturic mediators and it results in impaired energy metabolism, leading to cellular dysfunction in many organ systems. Adequate supplementation of phosphate with fluids containing potassium phosphate and oral/parenteral replacement are strongly recommended [118,119].

Postoperative pain control is of utmost importance for early mobilization and respiratory function. According to the ERAS guidelines, early mobilization after surgery should be encouraged from day one after surgery, taking for granted an effective multimodal approach to pain control [2,4,120].

## 8. Conclusions

Liver resection procedure is a high-complexity surgery with its inherent risks of postoperative adverse events (bleeding, respiratory complications, bile leaks and organ failure) that make perioperative management challenging for both anesthetists and surgeons.

Perioperative management of patients forwarded to hepatic resection of colorectal metastasis mainly targets hemodynamics stability, limitation of blood loss and blood products administration, preservation of metabolic balance both intra- and postoperatively and effective management of postoperative pain. All of these, along with potential surgical complications, make hepatic resection one of the highest-risk surgical procedures which may require postoperative stay in the critical care unit, at least in selected cases.

Thanks to the new era of fast-track surgery protocols, a safe outcome may be achieved if the following conditions are met: (1) the patient receives proper preparation to the surgery aimed at increasing his/her physiological reserve by chronic diseases’ assessment and clinical stabilization of them (nutritional status, preoperative evaluation, etc.); (2) the multidisciplinary team is sufficiently skilled to manage “hepatic surgery patients” (i.e., high-volume and referral centers); (3) integrated approach with a high degree of coordination of care, shared management plan and cooperation within the team between the anesthetist and the surgeon. Hence, it is out of doubt that each issue of hepatic surgery should be included in a protocol shared with the surgical team.

Finally, the success and the good outcome also depend on the total amount of hepatic resection procedures performed at the hospital center: the more hepatic patients are operated, the lower the risk of poor outcome because of the higher skill of the staff to manage such patients.

## Figures and Tables

**Table 1:** The Child–Pugh scoring system ^1^.

Parameter	Numerical Score		
	1	2	3
Ascites	None	Slight	Moderate to severe
Encephalopathy	None	Slight to moderate	Moderate to severe
Bilirubin (mg/dL)	<2.0	2–3	>3.0
Albumin (g/dL)	>3.5	2.8–3.5	<2.8
Prothrombin time (s)	1–3	4–6	>6

^1^ Total Child–Pugh score: A, 5–6 points; B, 7–9 points; C, 10–15 points.
